# Study on coal pulverization characteristics and gas desorption mechanism based on impact crushing experiment

**DOI:** 10.1016/j.heliyon.2024.e30800

**Published:** 2024-05-09

**Authors:** Xiang Fu, Qixuan Wu, Xuan Liu, Yifan Wang, Teng Chang

**Affiliations:** College of Mining, Liaoning Technical University, Fuxin, 123000, China

**Keywords:** Coal and gas outburst, Impact crushing, Coal powdering, Particle size distribution, Gas desorption

## Abstract

The coal's particle size distribution properties after pulverization and the gas desorption behavior driven by pulverization are of profound meaning to the study of coal and gas outburst mechanism. In this paper, based on the impact crushing experiment, the tectonic coal and primary coal are crushed under different impact energy conditions. After screening the broken coal, the particle size distribution law is analyzed, and the characterization function suitable for the particle size distribution of coal particles after crushing is determined. The relationship between crushing work and new surface area and fractal dimension of coal body is discussed. The consequences indicated that the mass proportion of tectonic coal below 0.074 mm particle size is much huger than that of raw coal. *G*-*S*, *R*–*R*, and fractal distribution model describe the best particle size distribution of the two coals in the scope of 0.074∼4 mm. The new surface area added increases with the crushing work, and the tectonic coal is 1.34–1.96 times that of the raw coal. The fractal dimension diminishes first and then increases with the crushing work ratio. In addition, the gas desorption amount of coal particles with different particle sizes after coal pulverization was measured, and a dynamic model suitable for coal pulverization-driven gas desorption was established, and the experimental results were verified. The research results of this paper can provide experimental and theoretical basis for the analysis of energy dissipation in coal and gas outburst.

## Highlights

• Coal pulverization characteristics and characterization function of particle size distribution after crushing.
• Crushing work ratio affects the change of fractal dimension of coal.
• The dynamic model of gas desorption driven by pulverized coal is established.
• The model is verified by experimental data.

## Graphical abstract

**Figure:**
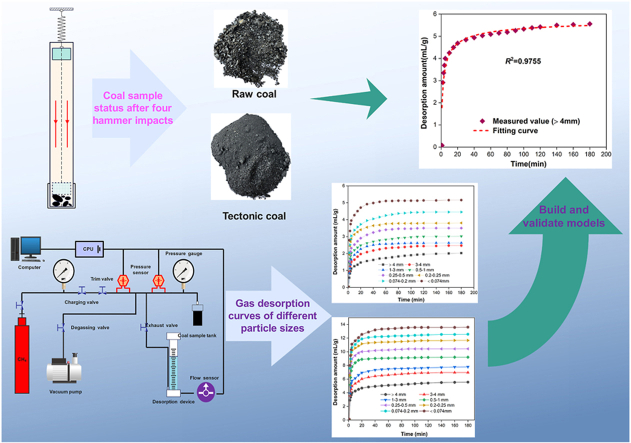

## 1 Introduction

The production process of coal mines involves a highly intricate, dynamic phenomenon known as coal and gas outburst, which pose a serious threat to coal mine safety [1,2]. Specifically, within a few seconds or tens of seconds of the outburst accident, a great number of coal and rock will be tossed toward the mining site, accompanied by hundreds or even millions of cubic meters of gas emissions. Based on scene data statistics, the outburst coal has canonical fragmentation, comprising a great number of granular coal or pulverized coal with particle size less than 1 mm [[3], [4], [5]]. The course of coal and gas outburst is also a procedure of rapid release of gas internal doublerun drill energy and coal crushing and throwing. The continuous crushing of coal body below the function of elastic potential energy accumulated in coal rock is a essential requirement for the occurrence and development of outburst [[6], [7], [8]]. Therefore, in-depth study of coal crushing related content is very important for understanding the mechanism of outburst, and it may similarly bring guiding value to assessment and prevention of coal mine disasters.

The particle size distribution and energy conversion in the crushing course are two main problems in the study of coal crushing. To explore the fragmentation of coal in the course of outburst, many researchers have discussed the fragmentation of coals from various angles. Wang et al. [9] believed that compared with pulverized coal, the energy required for tectonic coal outburst is significantly reduced. Jiang et al. [10] combined with coal powder adsorption and desorption experiments, considered that gas desorption can produce greater energy to apply to coal crushing. Jin et al. [11] deemed that the high velocity collision between particles and particles, particles and walls in the course of the outburst course will cause the coal to be crushed. Based on the rheological phenomenon of soft coal, Liu [12] proposed the concept of pulverized coal pool containing a great number of expansion energy and gas. Since B.B.Hodort put forward the crushing work of coal in the process of outburst, many scholars have put forward relatively accurate calculation methods of crushing power consumption based on area hypothesis theory, volume hypothesis theory and crack hypothesis theory [[13], [14], [15]]. Stamboliadis [16] introduced the particle size distribution law and found the analytical model of power consumption calculation. Bengtsson [17,18], Reddish [19] and Whittles [20] explored the connection between rock crushing power and particle size by numerical simulation and drop hammer experiment. Li et al. [21] used the particle extent fractal allocation model to construct a crushing kinetic equation to predict the particle extent allocation features in the coal crushing process. According to fractal rationale and particle extent allocation information, Zhang et al. [22] constructed the fractal model of particle extent of compacted and crushed coal. Zhou et al. [23] studied the crushing experiments of coal samples with two particle extents under five external pressures by using a hydraulic servo testing machine, and proposed models for predicting the particle extent allocation of coal particle crushing products. Zhang et al. [24] found that the double phase fluid of gas will break great particles into granulum while it is ejected from the outburst port at high velocity, and the crushing outcome rises with the increase of methane pressure. Based on the split Hopkinson pressure bar measurement system, Ding et al. [25] studied dynamic crushing tests on coals under different impacts, and studied the relationship between crushing load and particle extent of fragmented coal. Luo et al. [26] evaluated the impact power of coal with distinct particle extents in outburst. Wang et al. [27] analyzed various factors affecting coal particle breakage and found that apparent density of coal were significantly positively correlated with particle strength. Li et al. [28] consider the affect of methane pressure and adsorbed gas type on particle size distribution and crushing energy by drop hammer method, and found that balance pressure and gas pattern may influence fractal distribution parameters.

In addition, methane is another energy source of coal and gas outburst in addition to ground stress [29]. Relevant research shows that the operate of gas on outburst is mainly embodied in two sides. First, the presence of methane will change the mechanical properties of coal and rock. Second, the rapid desorption of gas will produce huge energy, provide power for the outburst sustainable development [30,31]. In the course of outburst, the free methane mainly participates in the outburst in the form of expansion work. When the external pressure is lower than the pore pressure of the coal, the free methane expands and destroys the coal body. The adsorbed gas adsorbs on the appearance of the coal pore, which reduces the surface energy of the coal, thus changing the mechanical properties of the coal body and reducing its mechanical strength. Unlike the work done by direct swelling of free methane, the adsorbed methane is not directly involved in the outburst. Instead, it is first quickly desorbed into free methane, and then involves in the work done by outburst with free gas after surface desorption, pore diffusion and fracture seepage [[32], [33], [34], [35], [36]].

Scholars have done a lot of research on the particle size distribution and energy consumption in the process of coal crushing. However, there is little research on the gas desorption mechanism of coal particles after different coal crushing. The research content is of great significance for revealing the gasification and desorption of gas containing pulverized coal in the process of coal and gas outburst. Therefore, this paper takes raw coal and tectonic coal as the research object, starting from the analysis of particle size distribution after crushing, studies the characteristics of particle size distribution of two coal, determines the characterization function suitable for particle size distribution after impact crushing of coal particles, and discusses the relationship between crushing work and new surface area and fractal dimension of coal body. This study can provide experimental basis and theoretical basis for energy dissipation analysis in outburst. Besides, the amount of methane desorption of coal particles with different particle extents after coal pulverization was measured by a self-developed experimental device for methane desorption of particles. The mechanism of gas desorption driven by coal pulverization was studied, and a kinetic model suitable for gas desorption driven by coal pulverization was established. The research findings offer a novel method for predicting coal and gas outburst risks and offer technical assistance for preventing and controlling outburst accidents.

## 2 Coal impact crushing test

### 2.1 Acquisition and fabrication of coal samples

**Fig. 1:**
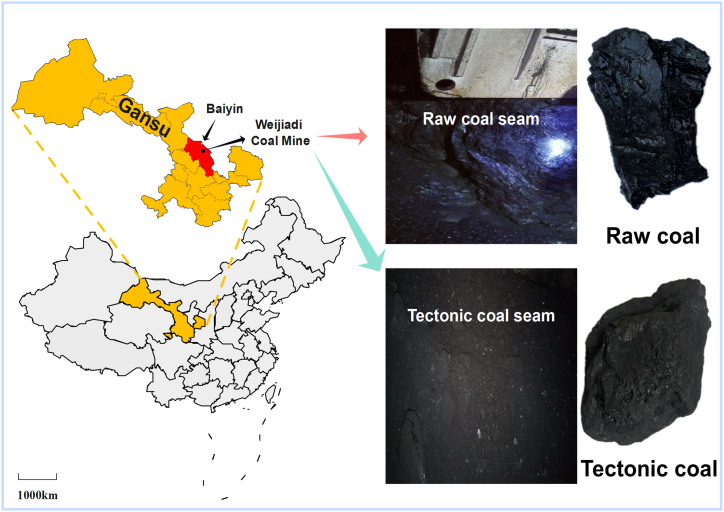
Collection locations of the coal samples.

In the experiment, the tectonic coal and raw coal from Weijiadi Coal Mine in Gansu Province, China were chosen. The large coal samples collected underground were crushed, and the lump coal of about 10 cm was selected for the impact crushing experiment. The sampling location is shown in Fig. 1. The firmness coefficient *f* of sample, the initial speed of methane emission Δ*p*, and the results of industrial analysis are displayed in Table 1.

**Table 1:** Foundation arguments for measurement of the coal samples.

Sampling location	Coal sample	*f*	Δ*p* (mmHg)	*M*_ad_ (%)	*A*_ad_ (%)	*V*_ad_ (%)	Coal type
Weijiadi Coal Mine	Tectonic coal	0.24	15.3	2.76	28.48	23.80	Bituminous coal
Weijiadi Coal Mine	Raw coal	0.92	10.5	1.55	43.68	18.71	Bituminous coal

### 2.2 Experimental apparatus and methods

**Fig. 2:**
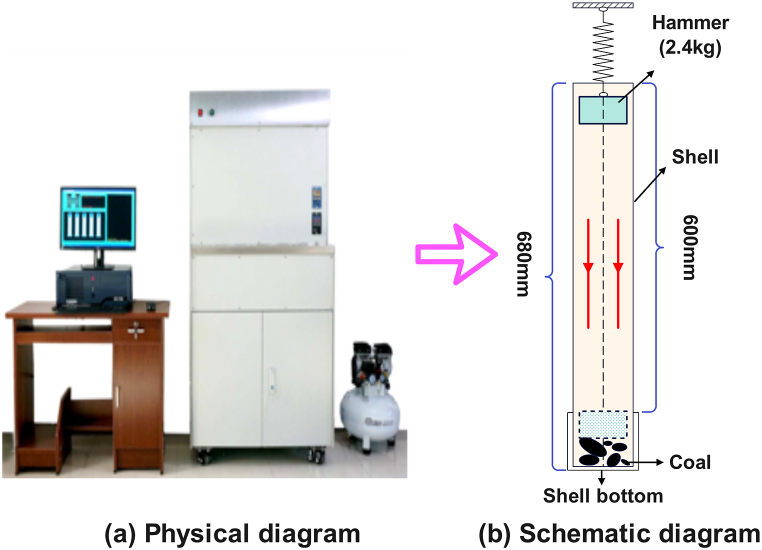
Coal firmness coefficient tester: (a) physical diagram; (b) schematic diagram.

The coal crushing experiment uses the impact crushing method to crush the collected coal samples. Impact crushing has the superiorities of large crushing ratio and low power consumption, and has become the main method for crushing lump coal. In this experiment, the FA-5 type firmness coefficient intelligent measuring instrument is used. The experimental apparatus can set the number of shocks arbitrarily after improvement, which reduces the experimental error caused by human factors. It has the superiorities of simple operation and accurate results, as displayed in Fig. 2. In the laboratory, tectonic coal and raw coal blocks with an initial particle size of 10 cm were selected for impact crushing tests. The quality of sample was 100 g. The same sample was repeated four times, and each time was measured. The coal sample was screened with a sample sieve. The sample sieve was used to meet the standard GB/T 60031-1997, and then the quality of pulverized coal in each interval was weighed with a balance.

Based on the new surface theory, the internal of coal smashing is that the coal rock mass obtains power and produces a new surface [9,11,12,37]. The physical meaning of crushing work ratio refers to the quantity of crushing work used up by the new surface area per unit area formed by coal in the crushing course [28,29,34].

The calculation of crushing work is displayed in Eq. (1) [28],

(1) $$\begin{array}{c} W=mg_{n}hn \end{array}$$

where *W* is the crushing work, J; *m* is the weight of hammer, kg; *g*_n_ is the acceleration of gravity, m/s^2^; *h* is the lifting height of hammer, m; *n* is the number of impacts.

Assuming that the coal particles are standard spheres, the new surface area during the crushing process of coal particles may be obtained according to the particle size distribution data obtained after crushing [[28], [29], [30]],

(2) $$\begin{array}{c} \Delta S=S_{i}-S_{0} \end{array}$$

(3) $$\begin{array}{c} \Delta S=\sum \frac{6m\gamma_{i}}{\rho d_{i}}-\frac{6m}{\rho d} \end{array}$$

where △*S* is the new surface area in the crushing process, m^2^; *m* is the total mass of coal sample, kg; *γ*_i_ is the quality ratio of coal particles in a certain particle extent scope after crushing, %; *ρ* is the density of coal sample, kg/m^3^; *d*_j_ is the mean value of coal particle size in a definite particle extent scope after crushing, m; *d* is the mean value of coal particle extent before crushing, m.

Based on total crushing work and new surface area obtained by the above calculation, the crushing work ratio may be further obtained [29],

(4) $$\begin{array}{c} \Gamma =\frac{W}{\Delta S} \end{array}$$

where *Γ* is the specific work of crushing, J/m^2^.

### 2.3 Test outcomes and discussion

#### 2.3.1 Distribution law of tiny coal particles after crushing

**Fig. 3:**
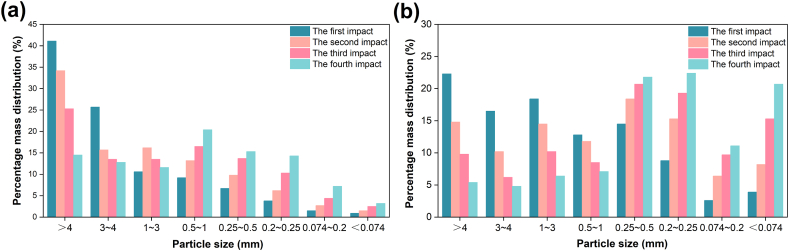
Mass ratio distribution of particle extent interval after the impact crushing experiment: (a) raw coal; (b) tectonic coal.

Fig. 3 displays the particle size distribution of raw coal and tectonic coal post-impact crushing. As the impact times add, the mass of coal particles with larger particle size gradually decreases, while the quality of particles with smaller particle extent gradually increases. The mass proportion of tectonic coal below 0.074 mm particle size is larger than raw coal. Compared with raw coal, the mass distribution of particles in tectonic coal is more uniform.

To quantitatively describe the features of coal under impact crushing, according to the results of particle extent distribution analysis (Fig. 3), the corresponding distribution function is used to analyze the particle extent distribution. Particle size distribution is usually subdivided into particle number distribution and mass distribution, which refer to the number percentage and mass percentage of particles, respectively. In most studies, because the number of particles is far more difficult than the mass, the mass distribution is generally used. At present, *G*-*S* (Gaudin-Schuhmann) distribution, *R*–*R* (Rosin-Rammler) distribution, fractal particle size distribution and lognormal distribution are commonly used to refer the particle extent distribution [[38], [39], [40]].

Gandin-Schuhmann distribution,

(5) $$\begin{array}{c} y={\left(\frac{x}{k}\right)}^{\tau } \end{array}$$

where *y* is the percentage under sieve of particle extent *x*; *x* is particle extent, mm; *k* is the distribution characteristics of particle extent. When *x* = *k*, the sieve quantity is 100 %, so *k* is the maximum particle extent. *τ* is the particle extent distribution index, determined by distribution characteristics.

Rosin-Rammler distribution,

(6) $$\begin{array}{c} y=1-\exp\left[-{\left(\frac{x}{b}\right)}^{a}\right] \end{array}$$

where *a* and *b* are constants associated to the distribution characteristics.

Fractal particle size distribution,

(7) $$\begin{array}{c} y={\left(\frac{x}{x_{m}}\right)}^{3-D_{f}} \end{array}$$

where *x*_m_ is the maximum particle extent, mm; *D*_f_ is the dimension of particle extent distribution.

Lognormal distribution holds that the distribution density of particle extent is normal while the particle extent is log-coordinated. The frequency of the distribution is represented by *φ*(ln *x*), then the distribution of particle extent is displayed in Eq. (8),

(8) $$\begin{array}{c} \varphi \left(\ln\;x\right)=\frac{1}{\sqrt{2\pi \sigma }}\exp\left[-\frac{{\left(\ln\;x-\ln\;x_{a}\right)}^{2}}{2\sigma^{2}}\right] \end{array}$$

where *x*_a_ is the geometric mean of grain size, mm.

The *x*_a_ can be obtained by Eq. (8),

(9) $$\begin{array}{c} \ln\;{\;x}_{a}=\frac{\sum_{i=1}^{i=n}\left(\gamma_{i}\;\ln\;x_{i}\right)}{\sum_{i=1}^{i=n}\gamma_{i}} \end{array}$$

where *γ*_i_ is the quality ratio of coal particles in a definite particle extent scope after crushing, %.

Therefore, the cumulative amount under the sieve,

(10) $$\begin{array}{c} y=\int_{0}^{\ln\;x}\varphi \left(\ln\;x\right)d\left(\ln\;x\right) \end{array}$$

By analyzing Eqs. (10), (5), (6), (7), (8), (9), it can be found that the expressions of *G*-*S* distribution and fractal distribution are similar. When *τ* = 3-*D*_f_, the expressions are the same. And when *y* is small, the results of *G*-*S* distribution and *R*–*R* distribution are the same. This is because the function expression of *R*–*R* distribution is expanded by series, and then after the second term is discarded, *a* ≈ *τ*, *b* ≈ *k* can be obtained.

**Fig. 4:**
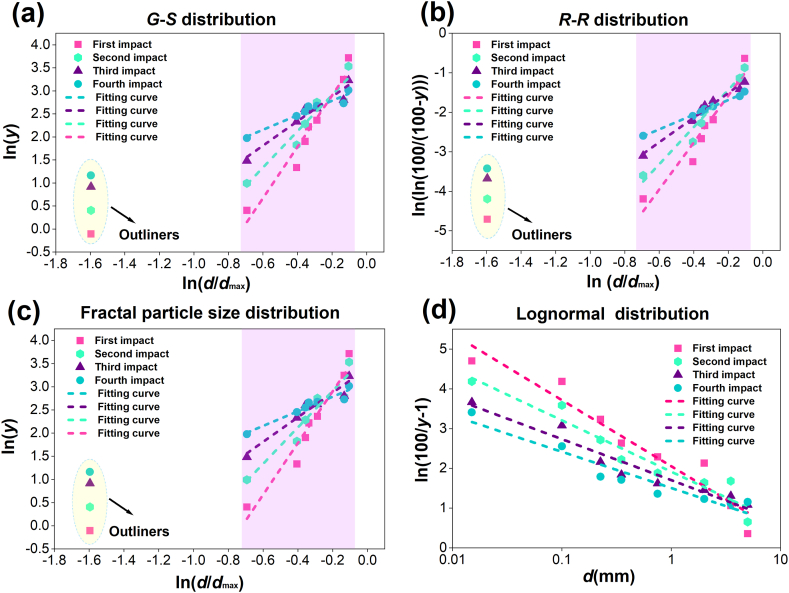
Linear fitting diagram of particle extent distribution models of tectonic coal under various impact times.

**Fig. 5:**
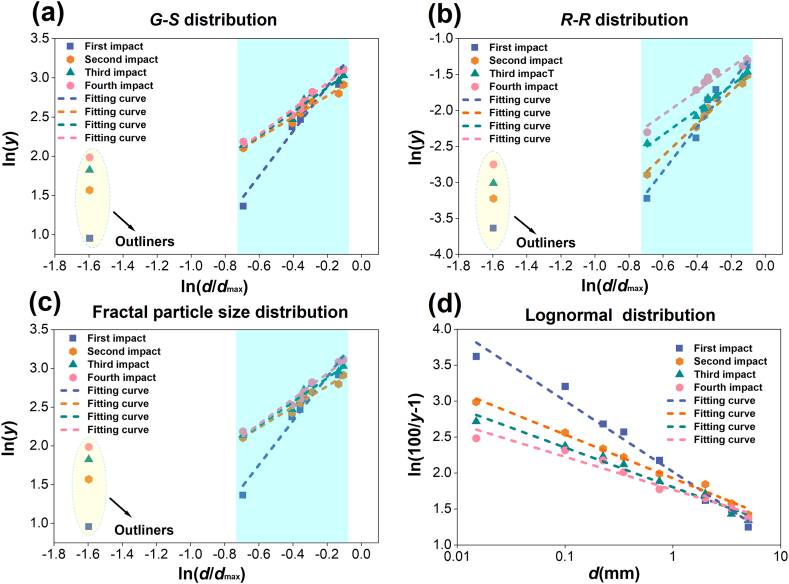
Linear fitting diagram of particle extent distribution models of raw coal under various impact times.

It can be seen from the above that the particle extent distribution of samples after crushing is influenced by the number of impact times. Now the correlation between the number of impact times and the particle extent distribution of coals after crushing is analyzed in detail. The particle extent distribution maps of tectonic coal and raw coal under different impact times are drawn respectively as shown in Fig. 4, Fig. 5.

From Fig. 4, Fig. 5, the *G*-*S* distribution model and fractal distribution model have the same linear fitting diagram, because the granularity distribution expressions of the two models are the same. It is displayed in the Figure that the particle extent distribution of coals broken under various impact times is similar, and the particle extent distribution of broken coal in a certain range conforms to each particle size distribution model. The *G*-*S* distribution, *R*–*R* distribution and fractal distribution model refer the optimal particle extent distribution of tectonic coal in the range of 0.074∼4 mm, and the optimal particle extent distribution of raw coal in the scope of 0.074∼4 mm. The part with particle size greater than 4 mm does not conform to the three particle extent distribution models with linear fitting. Therefore, the point of the part with particle size greater than 4 mm is defined as an abnormal value. The optimal particle size distribution range of lognormal distribution model for tectonic coal and raw coal is 0.074∼7 mm. Linear fitting is performed on the parts that fit well with the above four distribution models. The fitting outcomes under various impact times are displayed in Table 2. The fitting part has a good linear relationship, which indicates that the particle extent distribution model accurately describes the distribution law within the particle extent range.

**Table 2:** Linear fitting of each distribution model of tectonic coal and raw coal.

Coal types	Distribution model	Impact times	Linear fitting	*R*^2^
Tectonic coal	*G*-*S* distribution	1	ln(*y*) = 1.57ln(*d*/*d*_max_)+3.11	0.9154
		2	ln(*y*) = 5.57ln(*d*/*d*_max_)+4.02	0.9439
		3	ln(*y*) = 2.65ln(*d*/*d*_max_)+3.41	0.9241
		4	ln(*y*) = 3.93ln(*d*/*d*_max_)+3.69	0.9011
	*R*–*R* distribution	1	ln(ln(100/(100-y))) = 5.94ln(*d*/*d*_max_)-0.39	0.9320
		2	ln(ln(100/(100-y))) = 4.71ln(*d*/*d*_max_)-0.49	0.9787
		3	ln(ln(100/(100-y))) = 3.12ln(*d*/*d*_max_)-0.89	0.9565
		4	ln(ln(100/(100-y))) = 1.86ln(*d*/*d*_max_)-1.32	0.9949
	Fractal particle size distribution	1	ln(*y*) = 1.57ln(*d*/*d*_max_)+3.11	0.9154
		2	ln(*y*) = 5.57ln(*d*/*d*_max_)+4.02	0.9439
		3	ln(*y*) = 2.65ln(*d*/*d*_max_)+3.41	0.9241
		4	ln(*y*) = 3.93ln(*d*/*d*_max_)+3.69	0.9011
	Lognormal distribution	1	ln(100/*y*-1) = -1.66*d +* 2.05	0.9196
		2	ln(100/*y*-1) = -1.29*d +* 1.92	0.9189
		3	ln(100/*y*-1) = -1.03*d +* 1.71	0.9259
		4	ln(100/*y*-1) = -0.91*d +* 1.51	0.9041
Raw coal	*G*-*S* distribution	1	ln(*y*) = 2.85ln(*d*/*d*_max_)+3.46	0.9454
		2	ln(*y*) = 1.34ln(*d*/*d*_max_)+3.04	0.9488
		3	ln(*y*) = 1.54ln(*d*/*d*_max_)+3.18	0.9620
		4	ln(*y*) = 1.63ln(*d*/*d*_max_)+3.26	0.9664
	*R*–*R* distribution	1	ln(ln(100/(100-y))) = 3.07ln(*d*/*d*_max_)-1.01	0.9282
		2	ln(ln(100/(100-y))) = 2.46ln(*d*/*d*_max_)-1.29	0.9778
		3	ln(ln(100/(100-y))) = 1.71ln(*d*/*d*_max_)-1.31	0.9750
		4	ln(ln(100/(100-y))) = 1.65ln(*d*/*d*_max_)-1.06	0.9332
	Fractal particle size distribution	1	ln(*y*) = 2.85ln(*d*/*d*_max_)+3.46	0.9454
		2	ln(*y*) = 1.34ln(*d*/*d*_max_)+3.04	0.9488
		3	ln(*y*) = 1.54ln(*d*/*d*_max_)+3.18	0.9620
		4	ln(*y*) = 1.63ln(*d*/*d*_max_)+3.26	0.9664
	Lognormal distribution	1	ln(100/*y*-1) = -0.98*d +* 2.02	0.9753
		2	ln(100/*y*-1) = -0.61*d +* 1.93	0.9874
		3	ln(100/*y*-1) = -0.55*d +* 1.81	0.9743
		4	ln(100/*y*-1) = -0.46*d +* 1.77	0.9525

**Fig. 6:**
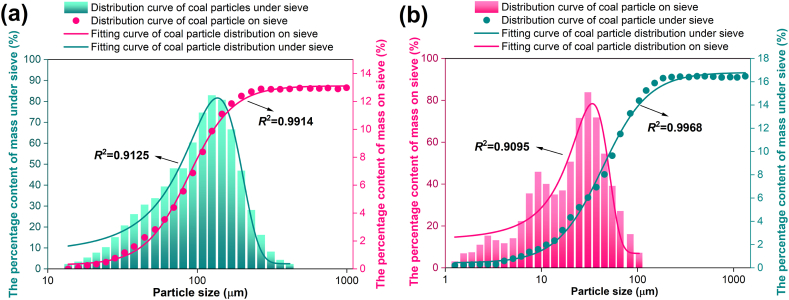
Micro particle distribution of coal post-impact crushing experiment: (a) raw coal; (b) tectonic coal.

Fig. 6 is the test outcomes of the analysis of the raw coal and tectonic coal after crushing the particle extent beneath 0.074 mm. The particle distribution law of raw coal obtained by data fitting shows that the mass distribution on the sieve obeys the lognormal distribution, as shown in Eq. (11) [38],

(11) $$\begin{array}{c} \varphi \left(\ln\;x\right)=\varphi \left(\ln\;x_{a}\right)+\frac{A}{\sqrt{2\pi }\omega x}\exp\left\{-\frac{{\left(\ln\;x-x_{a}\right)}^{2}}{{2\omega }^{2}}\right\} \end{array}$$

where *x* is the particle extent of coals, μm; *x*_a_ is the geometric mean of particle size, μm; *A*, *ω* are constants.

The cumulative mass distribution under the sieve obeys Eq. (12) [38],

(12) $$\begin{array}{c} y=A_{2}+\frac{A_{1}-A_{2}}{1+{\left(x/x_{a}\right)}^{p}}=A+\frac{C}{x_{a}^{p}+x^{p}} \end{array}$$

where *y* is the percentage under sieve of particle extent *x*; *A*, *A*_1_, *A*_2_, *C*, *p* are constants.

In Fig. 6(b), the quality distribution on the particle sieve of tectonic coal after crushing obeys the Gauss distribution, as displayed in Eq. (13) [38],

(13) $$\begin{array}{c} \varphi \left(x\right)=\varphi \left(x_{a}\right)+\frac{A}{\sqrt{\frac{\pi }{2}\omega }}\exp\left\{-\frac{2{\left(x-x_{a}\right)}^{2}}{\omega^{2}}\right\} \end{array}$$

The cumulative mass distribution of the particles under the sieve after the crushing of tectonic coal obeys Eq. (13), and the correlation coefficient is 0.9968.

In Fig. 6, the particle extent distribution of coal particles under the sieve is concentrated around 130 μm, and the mass percentage of particles with a particle extent of about 130 μm is the largest. The particle extent of raw coal is evenly distributed around the symmetry axis of 130 μm, while the particle extent of tectonic coal is primarily spread on the left side of 130 μm, that is, the quality percentage of tectonic coal particle extent less than 130 μm is more than raw coal. Evidently, the cumulative mass distribution of tectonic coal and raw coal under the sieve of particles with particle size below 0.074 mm is the same, while the distribution of mass percentage content on the sieve is different, one obeys Gauss distribution and the other obeys normal distribution. The same quality of tectonic and raw coal, under the condition of applying same energy, the tectonic coal is broken finer and the surface area is larger. Therefore, the two kinds of coal can be identified to a certain extent from the content distribution law on the sieve.

#### 2.3.2 The relationship between impact times and crushing work, new surface area and crushing work ratio

**Fig. 7:**
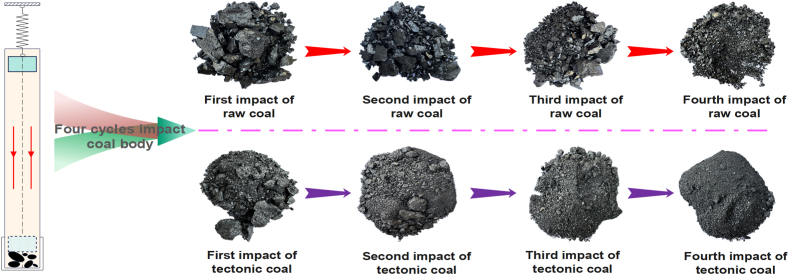
Distribution of coal particle state in impact crushing experiment of tectonic and raw coal.

Fig. 7 is the state of tectonic and raw coal after each crushing in impact crushing experiment. With the increase of impact times, the coal body is generally broken, and the extent of fragmentation of tectonic coal is much better than raw coal. Through Eqs. (1), (2), (3), (4), the new surface area and crushing work ratio of the four impact crushing of tectonic coal and raw coal can be obtained, as displayed in Table 3. In Table 3, the new surface area of raw and tectonic coal adds with the of impact times, and the new surface area generated by the fourth impact is the largest. The new surface area of tectonic coal after experiment is as high as 0.0587–0.5725 m^2^, which is 1.34–1.96 times that of raw coal (0.0437–0.2925 m^2^). The crushing work ratio is not linear relationship with impact times. With the increase of impact times, the crushing work ratio of raw coal and tectonic coal increases first then decreases. The reason for this phenomenon is that there is a certain limit to the crushing of coal. Another possibility is that when the impact number is large, the coal has been broken into smaller particle size, the gap between the particles is reduced, the work of overcoming the resistance between the particles during the drop hammer is increased, and the part of the crushing work consumed in the internal energy is increased, so that the crushing work is not increased. The crushing work ratio of raw coal and tectonic coal is between 193.18 and 323.26 J/m^2^ and 98.39–240.65 J/m^2^, respectively. It can be inferred from this that tectonic coal is more easily broken when given the same energy.

**Table 3:** Crushing work, newly added surface area, and crushing work ratio of coal samples.

Coal types	Impact times	Total crushing work (J)	Newly added surface area (m^2^)	Crushing work ratio (J/m^2^)
Raw coal	1	14.1264	0.0437	323.2586
	2	28.2528	0.0675	418.5600
	3	42.3792	0.0958	442.3716
	4	56.5056	0.2925	193.1815
Tectonic coal	1	14.1264	0.0587	240.6542
	2	28.2528	0.0925	291.0659
	3	42.3792	0.1456	305.4357
	4	56.5056	0.5725	98.3997

#### 2.3.3 Research of the relevance between crushing work and new surface area and crushing work ratio

**Fig. 8:**
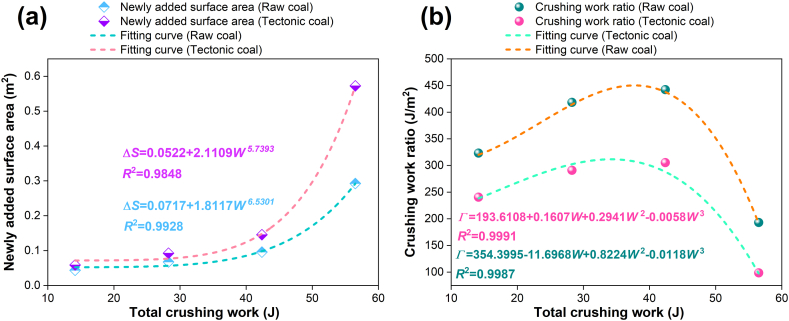
Relationship between total crushing work and newly added surface area, crushing work ratio: (a) newly added surface area; (b) crushing work ratio.

On the basis of the impact crushing experiment, the new surface area of the coal sample will change under the impact of each heavy hammer. Fig. 8 is the relevance between crushing work and new surface area, crushing work ratio. In Fig. 8(a), the new surface area of tectonic coal and raw coal increases with the increase of crushing work, and there is a certain functional relationship. Under the condition of applying the same energy, the new surface area of tectonic coal is greater than raw coal. In Fig. 8 (b), the crushing work ratio of tectonic and raw coal increases first and then decreases with increase of crushing work. The reason may be that when the number of impacts is large, the coal has been broken into a smaller particle size, and the gap between the particles is reduced. The work of overcoming the obstruction between the particles adds during the drop hammer, and the part of the crushing work consumed in the internal energy increases, so that the crushing work increases little, and the new surface area increases while the crushing work ratio decreases. Under the same crushing energy, the tectonic coal is easier to break.

#### 2.3.4 Research of the relevance between crushing work and fractal dimension

The concentration of tiny fracture groups forms the macroscopic fracture of coal, and smaller fractures develop and aggregate into larger ones. This self-similar behavior will necessarily lead to the self-similar characteristics of coal sample size after fracture. According to the relevance between the particle extent and the crushing work after the coal is broken, the fractal dimension of the coal sample after each crushing can be calculated by the fractal method, as shown in Eq. (14) [40],

(14) $$\begin{array}{c} D_{i}=3-\left[\mathrm{lg}\left(\frac{M_{r}}{M}\right)-\mathrm{lg}\;r_{\alpha }\right] \end{array}$$

where *D*_i_ is the fractal dimension; *M* is the total quality of coals, kg; *M*_r_ is the quality of coal sample in particle extent range, kg; *r*_α_ is the equivalent particle extent length in the particle size range, m.

**Fig. 9:**
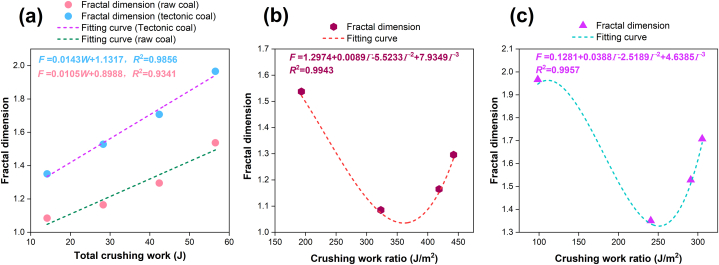
Relevance between total crushing work, crushing work ratio and fractal dimension: (a) total crushing work and fractal dimension of raw coal and tectonic coal; (b) crushing work ratio and fractal dimension of raw coal; (c) crushing work ratio and fractal dimension of tectonic coal.

Fig. 9 shows the relevance between crushing work, crushing work ratio and fractal dimension. In Fig. 9(a), the fractal dimension of tectonic coal and raw coal increases with the adds of crushing work. In Fig. 9(b) and (c), there is a cubic function relevance between the crushing work ratio and the fractal dimension. As the crushing work ratio increases, the fractal dimension initially reduces and subsequently grows.

In summary, the coal body is affected by the crushing work, which leads to the fragmentation fractal and then affects the gas desorption index, which is mainly reflected in the following aspects: 1. Coal crushing reduces its own particle size and increases its specific surface area; 2. Increase the pores and cracks inside the coal body; the most important point is that the tectonic coal is subjected to long-term high stress, and the micro-cracks are more developed. These cracks reduce the strength of the coal and make it more easily broken; that is, the crushing work is smaller.

## 3 Conditions of coal pulverization induced by methane action

During the transportation of coal in the mining space, the coal will be further pulverized due to particle collision and gas desorption. Considering that gas is the primary power source for coal handling, the pulverization of coal in this course is mainly due to gas action. In order to further study whether the coal will continue to pulverize below the action of methane, the conditions of raw pulverized coal caused by gas action will be analyzed theoretically.

### 3.1 The pulverization effect of coal particle collision

**Fig. 10:**
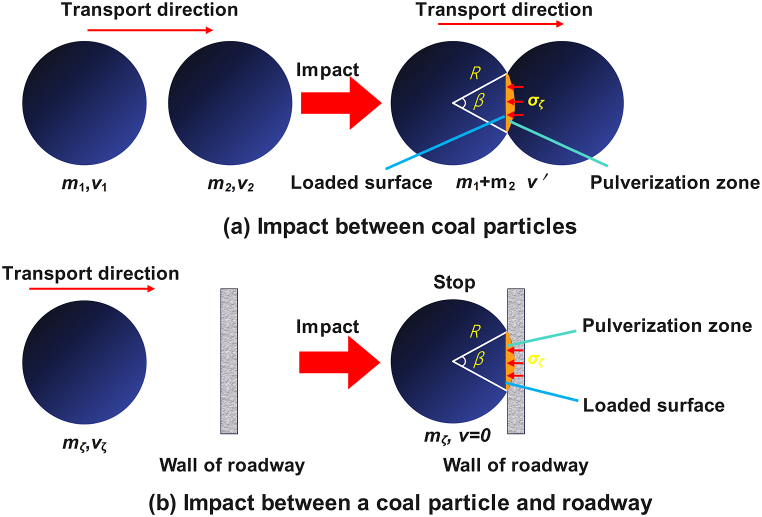
Diagram of impact course for coal particle: (a) impact between coal particles; (b) impact between coal particle and roadway.

In the course of coal handling, the collision between coal particles or with the roadway leads to local loading, resulting in the plastic destruction of the local coal at the collision spot, which will form the coal crushing. The collision between coal particles or with the roadway can be facilitated as displayed in Fig. 10. The contact area of the coal particles gradually grows and the contact surface stress gradually reduces as the coal body plastically fails close to the contact point. Local crushing will cease when the contact surface stress reaches the coal's compressive strength. Therefore, the spherical defect part of plastic failure is considered to be the crushing area of coal caused by particle collision, as shown in Fig. 10. The load on the contact surface during the collision process can be obtained according to the momentum theorem [41], as shown in Eqs. (15), (16),

(15) $$\begin{array}{c} F_{N}t_{\zeta }=\Delta P \end{array}$$

(16) $$\begin{array}{c} F_{N}=\sigma_{\zeta }\pi R^{2}\;\sin^{2}\left(\frac{\vartheta }{2}\right) \end{array}$$

where *F*_N_ is the contact surface load, N; *t*_ζ_ is the collision time, s; Δ*P* is the momentum vary of coal particles, kg·m/s; *σ*_ζ_ is the stress of the contact surface, MPa; *R* is the radius of coal particles, m; ϑ is the opening angle of the contact surface, °.

According to the circumstance of local crushing stop (*σ*_ζ_ = *σ*_c_), the opening angle of the final contact surface is gained by using Eqs. (15), (16), and the bulk of the crushing region (the ball part) can be indicated as Eq. (17) [41],

(17) $$\begin{array}{c} V_{\zeta }=\pi R^{3}{\left[1-\cos\left(\frac{\vartheta }{2}\right)\right]}^{2}\left[2+\cos\left(\frac{\vartheta }{2}\right)\right]/3 \end{array}$$

where *V*_ζ_ is the bulk of crushing region, m^3^.

Therefore, the crushing rate *η* of coal particles in the collision course may be calculated according to Eq. (18) [41],

(18) $$\begin{array}{c} \eta =\frac{4}{9}{\left[1-\cos\left(\frac{\vartheta }{2}\right)\right]}^{2}\left[2+\cos\left(\frac{\vartheta }{2}\right)\right]\times 100\% \end{array}$$

Next, the momentum change of coal particles before and after collision is discussed, as shown in Fig. 10(a). When particles collide, the condition of collision is that the velocity *v*_1_ of the rear particles is greater than the velocity *v*_2_ of the front particles. Assuming that the velocity $v^{＇}$ of the particles before and after the collision is the same, and ignoring the change of particle mass, the momentum change of a particle during the collision process can be calculated according to Eq. (19) [30,41],

(19) $$\begin{array}{c} \left\{ \begin{array}{c} m_{1}v_{1}+m_{2}v_{2}=\left(m_{1}+m_{2}\right)v^{＇}\\ \Delta P=\frac{m_{1}m_{2}\left(v_{1}-v_{2}\right)}{m_{1}+m_{2}} \end{array}\right. \end{array}$$

where *m*_1_ is the quality of rear coal particles, kg; *m*_2_ is the quality of coal particles in front, kg.

According to Eq. (19), the relative velocity difference between the two particles prior to contact determines the coal particles' change in momentum. Nevertheless, in the course of coal migration, the coal particles that collide with each other basically move in the same direction, and the speed of migration is very similar, which determines that the momentum change of coal particle collision is very small. When the particle collides with the roadway (barrier) (Fig. 10 (b)), the particle stops moving due to the influence of the barrier after the collision. Therefore, the momentum change of coal particles is,

(20) $$\begin{array}{c} \Delta P=\frac{4}{3}\pi R^{3}\rho v_{\zeta } \end{array}$$

where *v*_ζ_ is the velocity of coal particles, m/s; *ρ* is the density of coal, kg/m^3^.

Tu et al. [42], Jin et al. [43] believed that the migration velocity of coal is usually 10–50 m/s. Dong et al. [44] gained the compressive strength of coal particles in 1–3 mm particle size range is 2–5 MPa through coal particle compression experiment. According to the above data, the crushing rate of coal particles and roadway collision can be estimated by using Eqs (15), (18) and (20). The collision time *t*_ζ_ is 0.01 s, and radius of coal particles is 2–10 mm. The crushing rate is calculated to be 0.0015 %∼0.7214 %, so the crushing rate caused by the collision between coal particles and path is very low. The particle crushing caused by collision between particles is limited.

### 3.2 Grinding effect of gas desorption

During the transportation course, the consistence slope between the internal and external surfaces of the coal particles propels the methane desorption to diffuse from the internal to the external surface of the particles. The change of methane concentration leads to the change of gas pressure inside the coal particles, which makes the pressure gradient form the core to the outer surface of the coal particles.

Whether the gas desorption will cause the crushing of the raw coal particles depends on the methane pressure distinction between the inner and outer surfaces of the particles and the tensile strength of the particles. Wang et al. [45] considered that the methane pressure distinction between the inner and outer surfaces of the particles generally reduces with time, and the mrthane pressure distinction at the incipient time is the largest. Considering the influence of cracks and bedding planes, the tensile intensity of coal particles is far more than 1 MPa [42,44]. Furthermore, the coal particles have desorbed before stripping, and the internal methane pressure of particles in the initial stage of transportation has been greatly reduced compared with the primary methane pressure of coals. Therefore, the crushing of raw coal particles caused by gas desorption requires extremely high initial methane pressure conditions of coals.

Actually, the course of pulverized coal failure is the course of coal pore expansion and failure. On the one hand, the effect of pulverized coal increases the pore size, provides a more relaxed channel for gas diffusion, and increases the diffusion coefficient. Besides, the influence of pulverized coal makes the desorption of methane in coals and the desorption of methane in natural state have certain differences. Consequently, it is essential to discuss and build a gas desorption kinetic model suitable for pulverization influence on the basis of coal pulverization. The mechanism and course of gas desorption driven by pulverized coal will be further studied below.

## 4 Pulverized coal driven gas desorption mechanism and theoretical model establishment

### 4.1 Coal particle gas desorption experiment

**Fig. 11:**
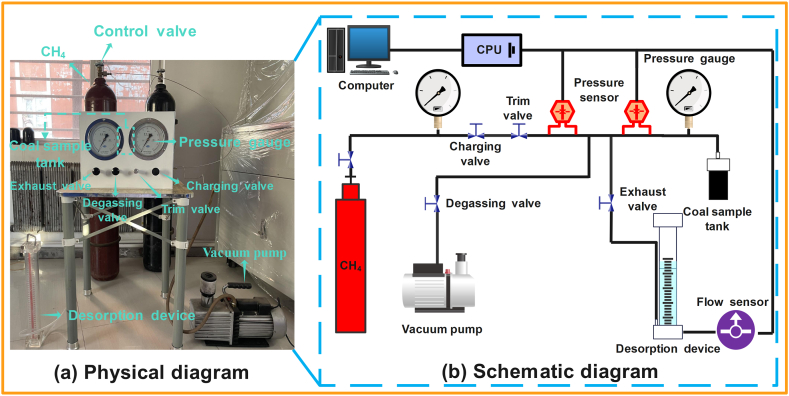
Coal particle methane desorption experimental instrument: (a) physical diagram; (b) schematic diagram.

The methane desorption amount of coal samples with distinct particle extents after impact crushing under the same adsorption equilibrium pressure was measured by the methane desorption experimental instrument displayed in Fig. 11.

### 4.2 Test result analysis

**Fig. 12:**
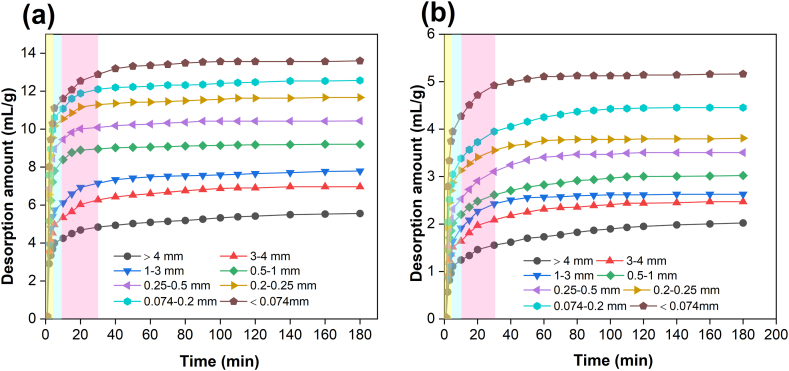
Methane desorption lines of coals with various particle extents: (a) tectonic coal; (b) raw coal.

The relevance curve between methane desorption amount and particle size of tectonic and raw coal is displayed in Fig. 12. The gas desorption lines of the two coals have the following main features. (1) The methane desorption curves of coal particles with distinct particle extents are similar, and the amount of gas desorption increases gradually with time. In the early stage, the methane desorption amount rises rapidly, and the line is steep. As time goes on, the desorption amount gradually decreases, and the desorption curve slows down. (2) The methane desorption curve of the coal sample with smaller particle extent is always above the coal sample with bigger particle extent, indicating that the methane desorption amount at same time adds with the decline of coal particle extent. (3) Belowr the same particle size, the methane desorption amount of tectonic coal is higher than raw coal, and this difference is more significant in the desorption curve of the first 5min.

Coal and gas outburst is a violent destruction course in a short time. It merely sustains for a few seconds or tens of seconds from excitation to termination [[46], [47], [48]]. For the whole process of coal and gas outburst, a lot of time is longer than this. But for a piece of coal, the most intense desorption is mainly in seconds to tens of seconds. Therefore, only the methane desorbed in a short time is effective for outburst, and the primary methane desorption features of coal are quite important to the formation of outburst.

The gas desorption amount (*Q*_1_,*Q*_2_,*Q*_3_) of tectonic and raw coal in the first 5min, the first 10min and the first 30min and its proportion in 180min gas desorption amount are statistically analyzed, as displayed in Table 4. It can be inferred from this that the methane desorption amount of tectonic coal and raw coal showed an increasing trend in the first 5 min, the first 10 min and the first 30 min. Compared with the original coal, the methane desorption amount of tectonic coal is larger at the primary time, and the methane desorption amount in the first 5 min is 2.82–4.09 times that of the original coal. Besides, the proportion of methane desorption in the first 5 min of tectonic coal is more than 14 %, indicating that tectonic coal has an exceedingly fast primary methane desorption ability, which can release the gas adsorbed by itself in a short time.

**Table 4:** Different particle size coal samples before 5 min, 10 min, 30 min gas desorption quantity and proportion.

Coal types	Datas	Coal sample size (mm)							
		＞4	3∼4	1∼3	0.5–1	0.25–0.5	0.2–0.25	0.074–0.2	＜0.074
Tectonci coal	*Q*_1_ (mL/g)	14.02	17.26	19.92	26.50	32.29	34.98	37.20	39.01
	Proportion (%)	14.66	14.16	14.46	15.49	16.48	16.09	16.45	15.61
	*Q*_2_ (mL/g)	18.27	22.61	26.04	34.89	41.75	45.52	48.28	50.61
	Proportion (%)	19.10	18.54	18.91	20.41	21.30	20.95	20.76	20.26
	*Q*_3_ (mL/g)	32.31	40.56	46.71	61.51	71.68	78.84	83.87	88.11
	Proportion (%)	33.76	33.27	33.92	35.97	36.57	36.28	36.07	35.27
Raw coal	*Q*_1_ (mL/g)	3.48	5.07	4.96	7.09	7.89	9.98	10.44	13.85
	Proportion (%)	11.09	12.35	11.08	13.70	13.03	14.58	13.51	14.79
	*Q*_2_ (mL/g)	6.04	6.71	6.88	9.29	10.41	13.12	13.82	18.12
	Proportion (%)	19.23	16.33	15.35	17.95	17.21	19.14	17.89	19.35
	*Q*_3_ (mL/g)	9.08	12.59	13.66	16.75	19.16	23.36	25.06	32.26
	Proportion (%)	28.93	30.62	30.49	32.35	31.66	34.07	32.44	34.46

**Fig. 13:**
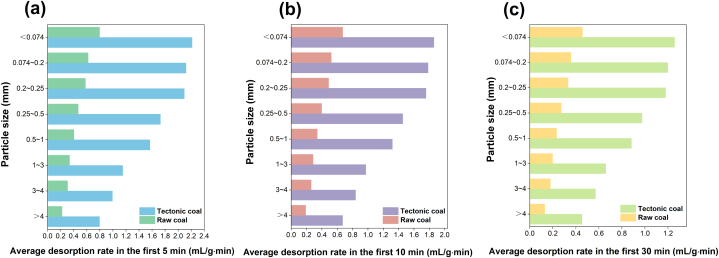
Relationship between primary methane desorption rate and coal particle size: (a) first 5min; (b) first 10min; (c) first 30min.

Further, the primary methane desorption rates of the two coal samples at distinct particle extents are quantified. Fig. 13 is the relationship between the equal desorption rate and particle size of tectonic and raw coal in the first 5 min, 10 min and 30 min. With the decrease of particle size, the initial methane desorption rate of tectonic and raw coal shows an increasing inclination. Among them, while the particle extent of coal is greater than 0.2–0.25 mm, the primary methane desorption rate of coal body increases greatly. As time goes on, the primary methane desorption rate of coal body declines obviously, and the average desorption rate of tectonic coal in the first 5 min is about 1.19 times of the average desorption rate in first 10 min. Furthermore, the average methane desorption rate of tectonic coal in the first 5 min is 3.92 times that of raw coal.

According to the above analysis, due to the transformation of coal pore structure by impact crushing, the methane desorption characteristics of coal body are directly affected, resulting in a great difference between the methane desorption characteristics of tectonic coal and that of raw coal.

### 4.3 Mathematical model of methane desorption driven by pulverized coal

Because the methane desorption driven by pulverized coal is an unsteady process, Thimons et al. [49] obtained a mathematical model of homogeneous spherical methane diffusion in coal based on Fick 's second determination, as shown in Eq. (21) [35],

(21) $$\begin{array}{c} \frac{\partial C}{\partial t}=\frac{D}{{r_{1}}^{2}}\cdot \frac{\partial }{\partial r_{1}}\left({r_{1}}^{2}\frac{\partial C}{\partial r_{1}}\right)=D\left(\frac{\partial^{2}C}{\partial {r_{1}}^{2}}+\frac{2}{r_{1}}\cdot \frac{\partial C}{\partial r_{1}}\right) \end{array}$$

where *C* is adsorption consistence, kg/m^3^; *r*_1_ is the radius of coal particles, m; *D* is the diffusion coefficient, m^2^/s; *t* is the diffusion time, s.

There is a certain difference between the process of methane desorption driven by pulverized coal and the process of methane desorption in the natural state of coal. In the process of pore expansion, the pore size and pore volume increase, which makes the methane diffusion coefficient add and shortens the gas diffusion time and diffusion distance. Besides, due to the devastation of the pore, the pore surface is transformed into outer surface and the surface diffusion occurs, and the methane consistence on the outer surface of coal will change significantly. Hence, in the course of coal pulverization, the amount of methane desorption will increase.

Based on the above analysis, the dynamic change of the diffusion coefficient and the surface diffusion after the exposed surface of the inner surface of the coal pore are considered in the construction of the kinetic model of methane desorption driven by pulverized coal. The model is simplified as follows. (1) The desorption course of methane driven by pulverized coal accords with Fick 's diffusion law. (2) The methane desorption course driven by pulverized coal is an isothermal isobaric process. (3) The law of coal adsorption methane conforms to the Langmuir Equation. (4) The flow of methane in the process of pulverized coal follows the law of conservation of quality and continuity equation. (5) In the process of coal pulverization, the methane diffusion coefficient increases with pore failure time. (6) After pulverized coal, gas desorption occurs on the pore surface, and surface methane concentration changes, and the change rate is recorded as *κ*.

Let *u* = *Cr*, put into Eq. (16), and put into the primary circumstances and boundary circumstances of pulverized coal, the kinetic model of methane desorption driven by pulverized coal can be gained, as displayed in Eq. (22) [35],

(22) $$\begin{array}{c} \left\{ \begin{array}{c} \frac{\partial u}{\partial t}=D\left(t\right)\frac{\partial^{2}u}{\partial r^{2}}\;\\ u=0\;\left(r=0,t>0\right)\\ u=C_{1}=\left(1-\kappa \right)C_{0}\;\left(r=r_{0},t>0\right)\\ u=C_{0}\;\left(0<r<r_{0},t=0\right) \end{array}\right. \end{array}$$

where *C* is the mass consistence of methane diffusion, kg/m^3^; *r* is the diffusion path, m; *C*_0_ is the mass consistence of gas after initial adsorption equilibrium, kg; *C*_1_ is the mass concentration of gas on the surface of coal, kg/m^3^.

The solution of Eq. (22) shows that at *r* = *r*_0_, the amount of methane desorption under pulverized coal circumstances is shown in Eq. (23) [30,35],

(23) $$\begin{array}{c} Q_{t}=4\pi r_{0}^{2}\int_{0}^{t}-D_{0}e^{\delta t}{\left(\frac{\partial C}{\partial r}\right)}_{r=r_{0}}\;dt\; \end{array}$$

where *Q*_t_ is the methane desorption amount at time *t*, mL/g; *D*_0_ is the methane diffusion coefficient of coal particles at *r* = *r*_0_, m^2^/s; *δ* is the increasing coefficient of the diffusion coefficient, s^−1^.

In Eq. (23) [27,30],

(24) $$\begin{array}{c} {\left(-\frac{\partial C}{\partial r}\right)}_{r=r_{0}}={\left(-\frac{\partial \left(\omega \left(r,t\right)\right)}{\partial t}\right)}_{r=r_{0}} \end{array}$$

After simplification, Eq. (23) is transformed into Eq. (25) [30,42],

(25) $$\begin{array}{c} Q_{t}=8\kappa \pi r_{0}^{2}C_{0}D_{0}\int_{0}^{t}e^{\delta t}\sum_{n=1}^{\infty }e^{-\frac{D_{0}n^{2}\pi^{2}}{\delta r_{0}^{2}}e^{\delta t}}dt \end{array}$$

Eq. (26) can be obtained by integrating Eq. (25) [30],

(26) $$\begin{array}{c} Q_{t}=\frac{8\kappa r_{0}^{3}C_{0}}{\pi }\sum_{n=1}^{\infty }\left[\frac{1}{n^{2}}\left(e^{\frac{D_{0}n^{2}\pi^{2}}{\delta r_{0}^{2}}}-e^{-\frac{D_{0}n^{2}\pi^{2}}{\delta r_{0}^{2}}e^{\delta t}}\right)\right] \end{array}$$

When *t → ∞*, the maximum gas desorption amount is *Q*_∞_, that is [8,42],

(27) $$\begin{array}{c} Q_{\infty }=\frac{4}{3}\pi r_{0}^{3}{\kappa C}_{0} \end{array}$$

(28) $$\begin{array}{c} \frac{Q_{t}}{Q_{\infty }}=\frac{Q_{\infty }-Q}{Q_{\infty }}=1-\frac{Q}{Q_{\infty }} \end{array}$$

Therefore, the methane desorption rate can be obtained as displayed in Eq. (29) [30],

(29) $$\begin{array}{c} \frac{Q_{t}}{Q_{\infty }}=1-\frac{6}{\pi^{2}}\sum_{n=1}^{\infty }\left[\frac{1}{n^{2}}e^{-\frac{D_{0}n^{2}\pi^{2}}{\delta r_{0}^{2}}\left(1-e^{\delta t}\right)}\right] \end{array}$$

Eq. (29) is the analytical solution of the gas desorption kinetic model in the process of pulverized coal.

### 4.4 Verification of gas desorption kinetic model driven by pulverized coal

The determination of diffusion coefficient is generally based on Fick 's diffusion law. Through the change curve of methane desorption amount and desorption time, ln(1-*Q*_t_/*Q*_∞_) is linearly fitted with *t*, and then the diffusion coefficient is obtained [8,27,50].

In Eq. (29), *n* is simplified to be 1, then [8],

(30) $$\begin{array}{c} \ln\;\left(1-\frac{Q_{t}}{Q_{\infty }}\right)=\delta \frac{D_{0}\pi^{2}}{r_{0}^{2}}\cdot e^{\delta t}+\ln\frac{6}{\pi^{2}}-\frac{D_{0}\pi^{2}}{\delta r_{0}^{2}} \end{array}$$

Let $\ln\frac{6}{\pi^{2}}-\frac{D_{0}\pi^{2}}{\delta r_{0}^{2}}$ = *ξ,* $\delta \frac{D_{0}\pi^{2}}{r_{0}^{2}}$ = *ψ*, then [8],

(31) $$\begin{array}{c} \ln\;\left(1-\frac{Q_{t}}{Q_{\infty }}\right)=\psi e^{\delta t}+\xi \end{array}$$

By analyzing the relationship between ln(1-*Q*_t_/*Q*_∞_) and *t*, the gas diffusion coefficient *D*_0_ of coal particles can be obtained [27,50],

(32) $$\begin{array}{c} D_{0}=\frac{r_{0}^{2}\psi }{\delta \pi^{2}} \end{array}$$

**Fig. 14:**
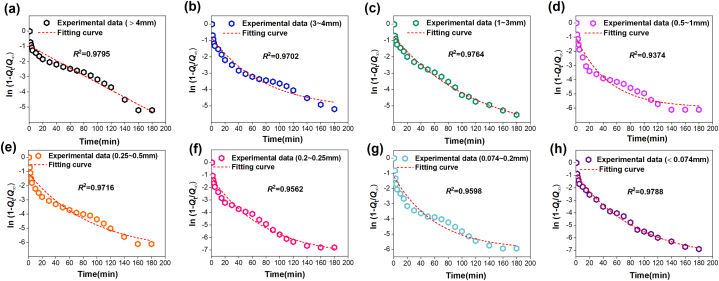
Methane diffusion coefficient and fitting curve in different particle size range during tectonic coal pulverization process.

**Fig. 15:**
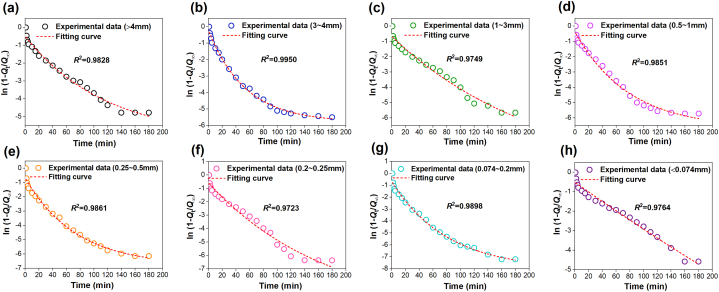
Gas diffusion coefficient and fitting curve in different particle size range during raw coal pulverization process.

According to the gas desorption amount measured in the experiment, the methane diffusion coefficient *D*_0_ and the increasing coefficient *δ* of the raw coal of tectonic coal under different particle extents may be calculated by regression analysis, as shown in Fig. 14, Fig. 15. The calculation results are displayed in Table 5.

**Table 5:** Gas diffusion coefficients are based on gas desorption experiment.

Coal types	Datas	Coal sample size (mm)							
		＞4	3∼4	1∼3	0.5–1	0.25–0.5	0.2–0.25	0.074–0.2	＜0.074
Tectonci coal	*D*_0_ (10^−9^ m^2^/s)	1.2671	1.2855	1.3025	1.3269	1.3508	1.3697	1.3857	1.4233
	*δ* (10^−4^ s^−1^)	1.98	2.14	2.29	2.42	2.65	2.81	2.99	3.12
	*R*^2^	0.9795	0.9702	0.9764	0.9374	0.9716	0.9562	0.9598	0.9788
Raw coal	*D*_0_ (10^−9^ m^2^/s)	1.0247	1.0445	1.0687	1.0798	1.0912	1.1125	1.1429	1.1822
	*δ* (10^−4^ s^−1^)	1.32	1.41	1.54	1.62	1.75	1.89	1.96	2.11
	*R*^2^	0.9828	0.9950	0.9749	0.9851	0.9861	0.9723	0.9898	0.9764

**Fig. 16:**
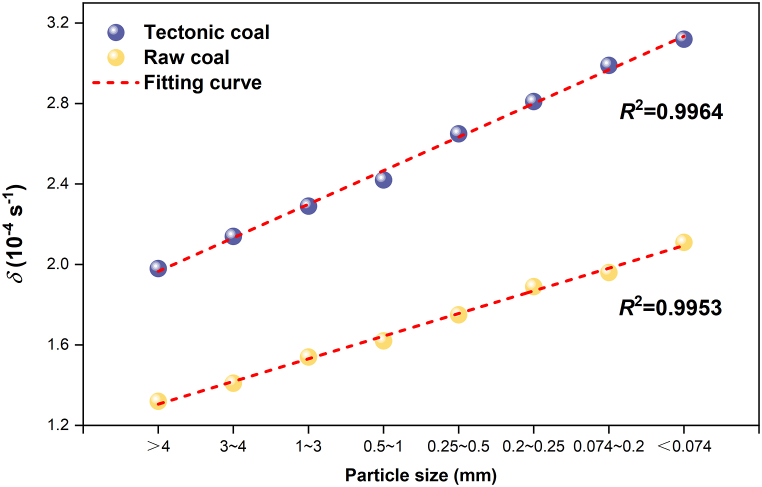
Relationship between the increasing coefficient of diffusion coefficient and the transform of coal particle extent.

Obviously, the diffusion coefficient *D*_0_ of the two coals adds with the decrease of particle size, and the diffusion coefficient of tectonic coal is much larger than raw coal in Figs. 14 and 15 and Table 5. The increasing coefficient *δ* of the diffusion coefficient also increases with the decline of particle extent. Fig. 16 shows the relevance between *δ* and coal particle extent. This is because as the number of crushing increases, the particle extent of the coal body decreases, the internal pores of coal body increase, easier the cracking and destruction of the coal pores, the more obvious the increase in pore size, and the pore bulk also changes. The room for methane molecular diffusion becomes larger and the diffusion capacity is enhanced, which ultimately increases the diffusion coefficient.

**Fig. 17:**
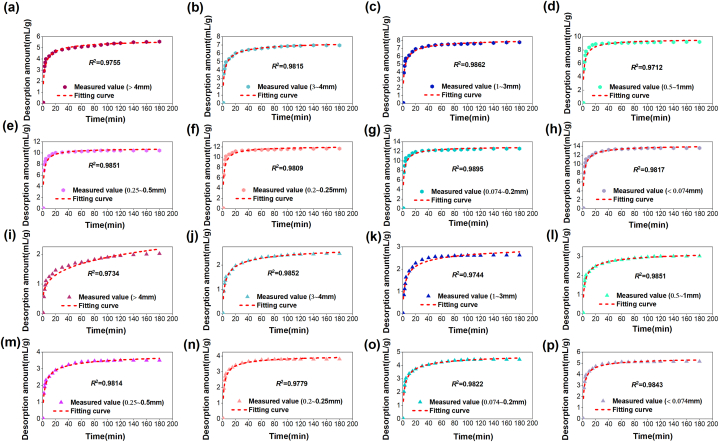
The theoretical model of methane desorption in the process of pulverized coal is fitting with the measured value: (a) tectonic coal; (b) raw coal.

According to the gas desorption data of two kinds of coals determined in Fig. 12, compared with the theoretical calculation results of the newly established pulverized coal-driven gas desorption kinetic model, as displayed in Fig. 17.

In Fig. 17, the theoretical calculation value of the methane desorption kinetic model in the process of coal pulverization caused by impact crushing is in good connection with the test value, and the correlation coefficient *R*^2^ is above 0.95. This displays that the kinetic model of methane desorption in the process of pulverized coal can reflect the process of gas desorption in the process of pulverized coal. In addition, the experimental data show that the model has a good application effect on the coals in the laboratory for methane desorption experiments and the phenomenon of pulverized coal caused by structural damage in the course of coal formation.

## 5 Conclusion

In this paper, aiming at the effect of the impact crushing characteristics of gas-bearing coal, the crushing experiments of tectonic coal and raw coal were carried out. The particle extent distribution of broken coal particles was analyzed, and the relationship between crushing work and new surface area and fractal dimension of coal was discussed. In addition, the gas desorption experiment was carried out on the coal particles, and the dynamic model of gas desorption driven by pulverized coal was constructed. The main conclusions are as follows.

(1) After the coal body was broken, the mass proportion of tectonic coal below 0.074 mm particle size is much larger than that of raw coal. The *G*-*S* distribution model, *R*–*R* distribution model, and fractal distribution model describe the best particle size distribution of the two coals in the range of 0.074∼4 mm.
(2) The new surface area added increases with the crushing work, and the tectonic coal is 1.34–1.96 times that of the raw coal. The fractal dimension decreases first and then increases with the crushing work ratio.
(3) The dynamic model of gas desorption driven by pulverized coal is established and verified. The results show that the measured gas desorption amount is in good agreement with the theoretical calculation value of the model, and the correlation coefficient *R*^2^ is above 0.95. The model can better reflect the methane desorption law in the process of pulverization.

## CRediT authorship contribution statement

**Xiang Fu:** Project administration, Funding acquisition, Conceptualization. **Qixuan Wu:** Supervision, Project administration, Data curation. **Xuan Liu:** Writing – review & editing, Writing – original draft, Investigation, Data curation. **Yifan Wang:** Writing – review & editing, Visualization. **Teng Chang:** Visualization, Supervision.

## Declaration of competing interest

The authors declare that they have no known competing financial interests or personal relationships that could have appeared to influence the work reported in this paper.
